# Toll-Like Receptors and Mannose Binding Lectin Gene Polymorphisms Associated with Cryptosporidial Diarrhea in Children in Southern India

**DOI:** 10.4269/ajtmh.20-0617

**Published:** 2021-09-27

**Authors:** Farzana Begum Liakath, Savitha Varatharajan, Prasanna Samuel Premkumar, Chanduni Syed, Honorine Ward, Gagandeep Kang, Sitara S. R. Ajjampur

**Affiliations:** ^1^ The Wellcome Trust Research Laboratory, Division of Gastrointestinal Sciences, Christian Medical College, Vellore, India;; ^2^ Division of Geographic Medicine and Infectious Diseases, Tufts Medical Center, Boston, Massachusetts

## Abstract

In low-resource settings, *Cryptosporidium* spp. is a common cause of diarrheal disease in children under the age of 3 years. In addition to diarrhea, these children also experience subclinical episodes that have been shown to affect growth and cognitive function. In this study, we screened polymorphisms in the promoter and exon1 regions of the mannose binding lectin 2 (*MBL2*) gene, as well as single nucleotide polymorphisms (SNPs) described in toll-like receptors (TLR) TLR1, TLR2, TLR4, and TLR9 and TIR domain-containing adaptor protein (*TIRAP*) genes among children with cryptosporidial diarrhea (cases) and children who only experienced asymptomatic (subclinical) cryptosporidiosis (controls). Among the polymorphisms screened, the variant allele B at codon 54 (rs1800450) of the *MBL2* gene was associated with susceptibility to cryptosporidial diarrhea (odds ratio [OR] = 2.2, 95% confidence interval [CI] 1.1–4.5). When plasma *MBL* levels were compared, 72% of cases were found to be deficient compared with 32% among controls (OR = 5.09). Among *TLR* polymorphisms screened, multivariate analysis showed that heterozygous genotypes of *TLR4* 896A/G (rs4986790, OR = 0.33, 95% CI: 0.11–0.98) and *TIRAP* 539 C/T (rs8177374, OR = 0.19, 95% CI: 0.06–0.64) SNPs were associated with protection from cryptosporidial diarrhea. Although not statistically significant, these findings suggest that polymorphisms of *MBL2* and *TLR* genes influence susceptibility to symptomatic cryptosporidial diarrhea even in settings with high exposure levels. Further studies to validate these findings in a larger cohort and to understand the role of these polymorphisms in mediating innate and adaptive immune responses to cryptosporidial infection are necessary.

## INTRODUCTION

Diarrhea is the second leading cause of morbidity and mortality in children under the age of 5 years in low- to middle-income countries.^1^ The Global Enteric Multi-Center Study and Malnutrition and Enteric Diseases studies as well as the Global Burden of Diseases estimates have identified *Cryptosporidium* spp. as one of the commonest causes of diarrhea in children under 5 years of age in low- to middle-income countries.^2^^–^^4^ *Cryptosporidium*-associated diarrhea alone accounts for more than 48,000 deaths and more than 4.2 million disability-adjusted life years lost per year.^3^ Children in countries such as Bangladesh, Peru, Malawi, Israel, and India also experience multiple episodes of subclinical or asymptomatic cryptosporidial infections that have been associated with malnutrition, stunting, and cognitive deficits.^5^^,^^6^ Birth cohort studies have shown that children in low-resource settings such as urban slums in the Indian subcontinent are exposed to *Cryptosporidium* spp. by age 3 years (97% in Vellore, Tamil Nadu, and 77% in Mirpur, Bangladesh), experiencing either diarrheal or subclinical episodes.^7^^,^^8^ The heterogeneity in symptoms and why some children tend to experience overt diarrhea and others only subclinical infections in the same living conditions remain largely unknown. This could be either due to parasite genotype or host susceptibility. Multiple infections (both symptomatic and subclinical) tend to cluster, with children with more than two or three episodes having an increased likelihood of being reinfected, suggesting a genetic predisposition to infection.^7^

The mannose binding lectin 2 (*MBL2*) gene encodes for a calcium-dependent plasma lectin that has been shown to play an important role in innate immune response to various infectious diseases. Low serum MBL levels (< 70 ng/mL) have been associated with cryptosporidial diarrhea in Haitian children.^9^ Carmolli et al. showed that children in Bangladesh who experienced both single and multiple episodes of cryptosporidial diarrhea were more likely to be MBL deficient (< 500 ng/mL, odds ratio [OR] = 4.1 and 7.5, respectively) compared with children who had no symptomatic infections. They also found that a single nucleotide polymorphism (SNP) in the promoter region of the *MBL2* gene (-221, rs7096206) was associated with susceptibility to cryptosporidial diarrhea (OR 4.0).^10^ Apart from *MBL2* polymorphisms, SNPs of toll-like receptor (TLR) family and signaling adaptor proteins have been shown to influence susceptibility to a variety of infectious diseases.^11^ These SNPs have also been described in the Indian population and are associated with susceptibility to tuberculosis, *Helicobacter pylori* and hepatitis C virus.^12^^–^^15^ Polymorphisms in the TIR domain-containing adaptor protein gene (*TIRAP*) required for TLR signaling^16^ have been associated with protection from tuberculosis^17^ and increased risk of malaria^18^ in Indians. Studies in cell culture and animal models suggest a role for TLR2 and TLR4 in early response to cryptosporidial infections. In cholangiocyte cell cultures, infection with *Cryptosporidium* resulted in selective recruitment of TLR2 and TLR4 with the production of IL8 and IL6 via the MyD88 pathway.^19^ TLR4 expression was also found to be regulated by parasite miRNA (*let-7i*). MyD88-deficient mice, interferon-γ knockout mice, and TLR4 knockout mice have all been shown not to clear cryptosporidial infections.^20^ However, to date, no studies have explored the role of TLR polymorphisms in susceptibility to cryptosporidial infections. In the present study, we sought to determine the association of TLR and MBL polymorphisms as well as plasma MBL levels with cryptosporidial diarrhea in children residing in a periurban slum area in southern India.

## METHODS

In a previous study, children enrolled in a birth cohort on rotaviral diarrhea (CRI birth cohort) were followed up for all diarrheal episodes from birth until age 3 years between 2002 and 2006 in a semiurban slum in Vellore, southern India.^21^ In this study, comparison of MBL and TLR polymorphisms among cases defined as children in the birth cohort who experienced cryptosporidial diarrhea under the age of 2 years^22^ and controls defined as children in the birth cohort who only experienced asymptomatic (subclinical) cryptosporidiosis under the age of 2 years^23^ was carried out. Among the 452 children followed up in the birth cohort, 39 children were identified as having cryptosporidial diarrhea and were categorized as cases and 96 children who did not experience any cryptosporidial diarrhea who were younger than age 2 years (ascertained by *SSU rRNA* PCR for *Cryptosporidium* spp. carried out on all diarrheal samples) were categorized as controls. Blood samples were collected from 36 of 39 cases and 82 of 96 controls, DNA was extracted with the QIAamp DNA blood mini kit (Qiagen Inc., Valencia, CA), and SNPs were genotyped using previously described methods (Supplemental Table 1).^37^^--^^41^ Promoter diplotypes for *MBL2* were classified as described previously as LY/LX, HY/LY, LY/LY, LX/LX, HY/LX, and HY/HY.^24^ Haplotype frequencies for *MBL2*, *TLR4*, and *TLR9* were calculated based on the expectation-maximization algorithm using Haploview 4.1 and those with frequencies > 2% were compared. Hardy-Weinberg equilibrium was tested using the GENHWCCI function in STATA 11.0 (StataCorp, College Station, TX). Plasma levels of functional MBL were measured by ELISA using the Human MBL ELISA test kit (HyCult Biotechnology). The samples were tested at a 1:20 dilution, and MBL levels were estimated by interpolated values with linear standard curves as per the kit protocol.

Distribution of baseline characteristics and frequencies of SNPs were compared between cases and controls using either χ^2^ tests or Fisher’s exact tests, as appropriate. Association of SNPs were evaluated using ORs and 95% confidence intervals (CIs), and analyses were adjusted for birth weight and number of diarrheal episodes by logistic regression to produce adjusted ORs. *P* values were adjusted (p-adj) for multiple comparison using the Benjamin-Hochberg adjustment. A receiver operating characteristic curve (ROC) analysis was used to determine the appropriate cutoff to define plasma MBL deficiency in this population and assess the association with MBL2 promoter diplotypes. The study protocol was approved by the Institutional Review Board of the Christian Medical College, Vellore, and written informed consent was obtained from parents of all children enrolled in this study.

## RESULTS

**Figure 1.**
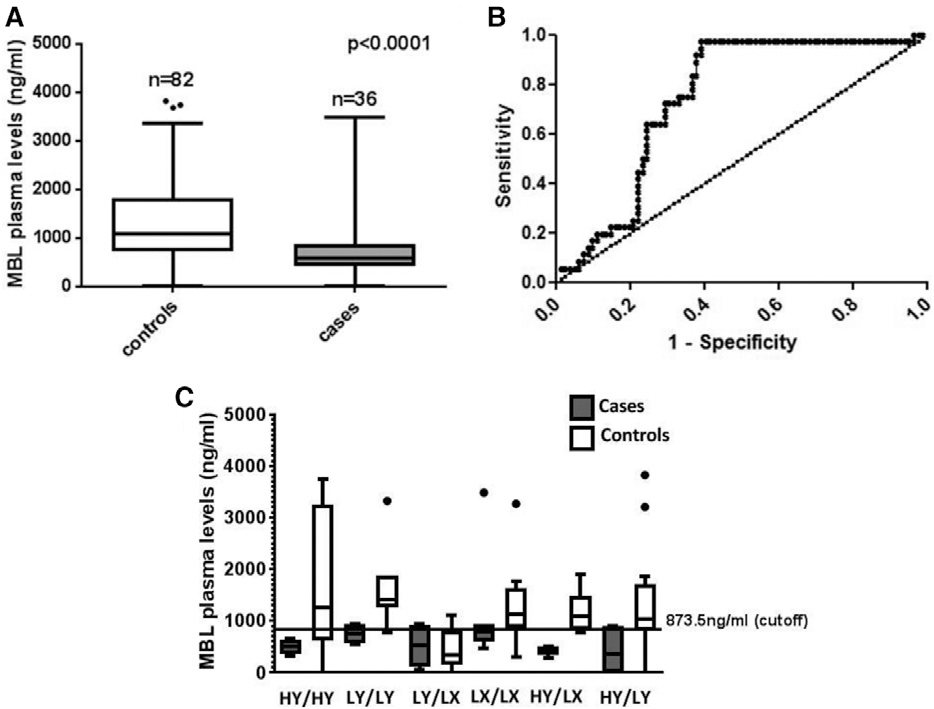
Plasma mannose binding lectin (MBL) levels in cryptosporidial cases and controls. (**A**) MBL levels between cases and controls. (**B**) Receiver operating characteristic analysis of MBL levels in controls to determine an optimal cutoff. (**C**) Comparison of MBL levels among the *MBL2* promoter diplotypes in cases and controls.

Comparison of baseline characteristics showed that more controls had low birth weight compared with cases (*P < *0.05) and cases had diarrhea more frequently (five to nine episodes) than controls (one to three episodes; *P < *0.001) (Supplemental Table 2).^42^ Hence, the multivariate logistic regression analysis performed to determine the association of SNP was adjusted for birth weight and the number of diarrheal episodes. Among the *MBL2* exon1 polymorphisms, the variant allele B at codon 54 (rs1800450) was found to be associated with susceptibility to cryptosporidial diarrhea (OR = 2.2, 95% CI: 1.1–4.5) (Table 1) in the multivariate analysis, whereas variant allele C at codon 57 (rs1800451) did not show any association (OR = 0.4, 95% CI: 0.1–1.6). Among *MBL2* haplotypes with a frequency > 2%, the HXBA haplotype was found to be associated with risk of cryptosporidial diarrhea (OR = 6.5, 95% CI: 1.0–41.3) (Table 2). However, when adjusted for multiple comparisons (p-adj), the *MBL2* rs1800450 polymorphism and HXBA haplotype association with cryptosporidial diarrhea was not significant. Plasma MBL levels ranged from 63.6 to 3823.2 ng/mL. The median age of children at which the MBL levels were measured was 5.91 years (range 5.3–6.7 years). Cases had significantly lower MBL levels (median 575.6 ng/mL, interquartile range [IQR] 420.3–883.2 ng/mL) than controls (median 1090.9 ng/mL, IQR 770–1824.7 ng/mL; *P* < 0.001) (Figure 1A). When plasma MBL levels among the controls for each promoter diplotype were compared, the LY/LX diplotype had markedly lower levels (*P* = 0.018) and was chosen to represent the “deficient” diplotype in this population. Receiver operating characteristic curve generated with plasma MBL levels in the “deficient” controls with the LY/LX diplotype showed that a cutoff of 873.5 ng/mL would be optimal (sensitivity 81%, specificity 77%, area under the curve = 0.83) (Figure 1B). When this cutoff was then reapplied to the diplotype distribution, all children (cases and controls) with the LY/LX genotype were classified as “deficient,” thereby validating the cutoff (Figure 1C). Using this cutoff, when the proportion of children with MBL deficiency among cases and controls was compared, more than twice the number of cases (72%) than controls (32%) were found to be deficient (OR = 5.09) (Figure 1C).

**Table 1:** Univariate and multivariate analysis of the association between *TLR*, *TIRAP*, and *MBL2* SNPs and cryptosporidial diarrhea

SNP	MAF %		Univariate			Multivariate adjusted only for birth weight				Multivariate adjusted for birth weight and number of diarrheal episodes			
	Cases, *n* = 36	Controls, *n* = 82	OR	95% CI	*P* value	OR	95% CI	*P* value	p-adj*	OR	95%CI	*P* value	p-adj*
*TLR4* rs4986790 896A/G Asp299Gly	11	23	0.38	0.16–0.90	0.08	0.31	0.12–0.76	0.01	0.04	**0.33**	**0.11–0.98**	**0.04**	0.16
*TLR4* rs4986791 1196 C/T Thr399Ile	11	18	0.54	0.22–1.31	0.38	0.44	0.18–1.09	0.08	0.22	0.45	0.15–1.37	0.13	0.36
*TIRAP* rs8177374 539C/T Ser180Leu	11	32	0.17	0.08–0.43	0.001	0.17	0.07–0.44	0.001	0.01	**0.19**	**0.06–0.64**	**0.007**	**0.08**
*TLR1* rs5743618 1805 T/G Ile602Ser	4	4	0.97	0.24–4.00	0.97	0.82	0.20–3.39	0.79	0.87	0.61	0.09–3.98	0.61	0.61
*TLR9* rs5743836 C-1237T	83	92	2.32	0.88–6.14	0.22	2.13	0.78–5.83	0.14	0.26	2.13	0.57–8.01	0.14	0.31
*TLR9* rs1870884 T-1486C	43	43	0.96	0.31–2.99	0.95	0.96	0.30–3.07	0.95	0.95	0.47	0.09–2.39	0.37	0.45
*TLR2* (-196 to -174) (del)	33	39	0.86	0.44–1.70	0.79	0.85	0.43–1.69	0.65	0.79	1.94	0.71–5.23	0.19	0.35
*MBL2* rs11003125 G-550C H>L	73	60	1.68	0.95–2.97	0.19	1.58	0.88–2.83	0.12	0.26	1.66	0.73–3.74	0.22	0.30
*MBL2* rs7096206 C-221G Y>X	33	27	1.26	0.74–2.17	0.59	1.17	0.66–2.07	0.58	0.79	0.80	0.54–3.62	0.48	0.53
*MBL2* rs1800450 230G/A Gly54Asp A>B	54	34	1.78	1.09–2.79	0.04	1.96	1.18–3.24	0.009	0.04	2.20	1.08–4.51	0.03	0.17
*MBL2* rs1800451 239 G/A Gly57Glu A>C	5	12	0.47	0.16–1.35	0.15*	0.47	0.16–1.35	0.16	0.25	0.43	0.11–1.61	0.21	0.32

CI = confidence interval; OR = odds ratio.

*p-adj = *P* value adjusted for multiple comparisons.

**Table 2:** Haplotype frequency of SNPs in *TLR4*, *TLR9*, and *MBL2* among children with and without cryptosporidial diarrhea

*TLR4* rs4986790 896A/G	*TLR4* rs4986791 1196 C/T	*TLR9* rs5743836 (-1237)	*TLR9* rs1870884 (-1486)	Controls	Cases	OR (95% CI)	*P* value	p-adj
G	C	C	T	0.36	0.54	1.00	–	
G	C	C	C	0.23	0.26	1.03 (0.29–3.68)	0.96	0.96
A	T	C	T	0.09	0.00	0.50 (0.12–2.03)	0.33	0.59
G	C	T	C	0.10	0.06	0.58 (0.10–3.30)	0.54	0.61
A	C	C	T	0.07	0.00	0.27 (0.05–1.37)	0.12	0.54
G	T	C	T	0.02	0.03	0.03 (0.06–1.55)	0.15	0.45
*MBL2* rs11003125 (-550)	*MBL2* rs7096206 (-221)	*MBL2* rs1800450 codon 54	*MBL2* rs1800451 codon 57					
C (L)	C (X)	G (A)	G (A)	0.26	0.08	1.00	–	
G (H)	C (X)	G (A)	G (A)	0.19	0.17	2.47 (0.40–15.45)	0.34	0.51
G (H)	G (Y)	G (A)	G (A)	0.17	0.21	1.78 (0.38–8.34)	0.47	0.60
G (H)	C (X)	A (B)	G (A)	0.15	0.08	**6.50** (1.02–41.27)	**0.05**	0.45
C (L)	C (X)	A (B)	G (A)	0.13	0.14	2.68 (0.49–14.65)	0.26	0.59

CI = confidence interval; OR = odds ratio.

*p-adj = *P* value adjusted for multiple comparisons.

When TLR polymorphisms were examined, the minor allele frequency (MAF) of the *TLR1* SNP rs5743618 was similar in cases and controls (Table 1), and all subjects enrolled in this study had the ancestral allele at the *TLR2* SNP rs5743708 locus, consequently neither of these SNPs was analyzed further. The *TLR4* SNP rs4986790 heterozygous genotype 896A/G and the *TIRAP* SNP rs8177374 heterozygous 539C/T genotypes were seen more often among controls than cases. Both SNP were associated with protection from cryptosporidial diarrhea in the multivariate analysis (OR = 0.33, 95% CI: 0.11–0.98, and OR = 0.19, 95% CI: 0.06–0.64, respectively) (Table 1) but only the *TIRAP* SNP approached significance (p-adj = 0.08). None of the other polymorphisms showed any significant association with cryptosporidial diarrhea. When haplotype analysis was carried out, no significant association for either *TLR4* or *TLR9* haplotypes was seen with the risk of cryptosporidial diarrhea (Table 2).

## DISCUSSION

Cryptosporidiosis remains an important cause of diarrheal disease and stunting in children in low-resource settings. In this study, genetic susceptibility to cryptosporidial diarrhea in an urban slum with poor sanitation and high levels of exposure was explored. Increased susceptibility to cryptosporidial diarrhea among those with plasma or serum MBL deficiency has been shown in Bangladeshi and Haitian children.^9^^,^^25^ Increased risk of cryptosporidial diarrhea has also been associated with *MBL2* polymorphisms in both the exon 1 region^25^^,^^26^ as well as the promoter region.^25^ Our study has further validated these findings in children in southern India. Plasma MBL2 deficiency and a SNP in the *MBL2* gene in the exon 1 region (codon 54) were associated with risk of cryptosporidial diarrhea. Because the samples collected were not temporally related to the episode of diarrhea, the levels of MBL estimated were not related to acute response but more reflective of baseline levels. Furthermore, the *MBL2* diplotype associated with low plasma MBL in the southern Indian population (LY/LX) was identified and a more appropriate cutoff to determine MBL deficiency was determined.

This is the first study to indicate an association between *TLR4* and *TIRAP* polymorphisms with cryptosporidiosis. Presence of the rs4986790 polymorphism in TLR4 has been found to have a protective effect in other intracellular pathogens as well, including *Mycobacterium lepra*^27^ and *Legionella*.^28^ In a more recent study by Ortega et al., the minor allele G was also associated with deceased risk of active tuberculosis (OR = 0.31).^29^ A meta-analysis of genetic susceptibility studies showed an increased odds of parasitic infections associated with this SNP, but none of the studies included in the analysis involved *Cryptosporidium* or any other gastrointestinal parasite.^30^ The *TIRAP* gene encodes an adaptor protein that is essential for downstream signaling of *TLR2* and *TLR4*.^31^ The nonsynonymous polymorphism in *TIRAP* SNP rs8177374 leads to altered binding of TIRAP to MyD88.^32^ Similar to the findings of this study, the *TIRAP* SNP has been associated with protection from invasive pneumococcal disease, bacteremia, malaria, and tuberculosis.^33^ The potential protective effect of these SNPs in cryptosporidial diarrhea could be due to decreased activation of downstream signaling pathways that lead to intestinal inflammation. Increased levels of inflammatory markers (including cytokines) have been found in stools of children with cryptosporidial diarrhea.^34^ These SNPs could also affect TLR mediation of adaptive immune response to cryptosporidiosis. Further mechanistic studies are required to determine the role of these SNP and whether they could serve as potential therapeutic targets.

In addition to MBL and TLR SNPs, SNPs in other genes have been shown to affect risk of cryptosporidial infections. The HLA class II DQB1*0301 allele was found to provide protective effect in Bangladesh children compared with symptomatic infection.^35^ More recently an intronic SNP (rs58296998) in the protein kinase C gene (*PRKCA*) identified by a genome-wide association study was found to be associated with higher risk of cryptosporidiosis in the first year of life among 873 children in Bangladesh.^36^ The authors in this study used samples collected from multiple, independent cohorts in Bangladesh to determine genetic susceptibility. A similar approach with samples from larger, well-characterized cohorts of children from the same genetic background are warranted to validate the findings of the present study. In conclusion, we have validated the association between MBL deficiency and increased risk of cryptosporidial diarrhea and identified SNPs, in *MBL2*, *TLR4*, and in *TIRAP* genes that may confer protection from developing diarrhea during a cryptosporidial infection.

## Supplemental Material


Supplemental materials
