# Supplementary material for: Toll-Like Receptors and Mannose Binding Lectin Gene Polymorphisms Associated with Cryptosporidial Diarrhea in Children in Southern India
Source: Am J Trop Med Hyg. 2021 Sep 27;105(6):1706–11. doi: 10.4269/ajtmh.20-0617 (PMC8641352; doi:10.4269/ajtmh.20-0617)
Supplement: Supplementary file 1 [file tpmd200617.SD1.docx]

**Supplemental Table 1: *TLR, TIRAP, and MBL2* Polymorphisms Analyzed in the study**

| **Gene** | **Chromosome location** | **SNP ID** | **Nucleotide position** | **Amino acid position** | **Method** | **Reference** |
| --- | --- | --- | --- | --- | --- | --- |
| ***TLR4*** | 9q32 | rs4986790 | 896 A/G | Asp299Gly | PCR-RFLP | ^37^ |
|  | 9q33 | rs4986791 | 1196 C/T | Thr399Ilr |  |  |
| ***TIRAP*** | 11q24.2 | rs8177374 | 539 C/T | Ser180Leu | PCR-RFLP | ^38^ |
| ***TLR1*** | 4p14 | rs5743618 | 1805 T/G | Ile602Ser |  |  |
| ***TLR9*** | 3p21.3 | rs5743836 | - 1237 C/T | Promoter |  |  |
|  | 3p21.3 | rs1870884 | - 1486 C/T | Promoter |  |  |
| ***TLR2*** | 4q31 |  | -196 to -174 | 22 bp deletion | Ins/Del PCR | ^39^ |
|  | 4q31.3 | rs5743708 | 2258 G/A | Arg753Gln | ARMS PCR | ^40^ |
| ***MBL2*** | 10q11.2 | rs11003125 | -550 G/C  H to L | Promoter | Allele specific PCR with 5’UTR internal control | ^41^ |
|  | 10q11.2 | rs7096206 | -221 G/C  Y to X | Promoter |  |  |
|  | 10q11.2 | rs1800450 | 230 G/A (codon 54)  A to B | Gly54Asp | Allele-specific PCR with human growth hormone (HGH) internal control |  |
|  | 10q11.2 | rs1800451 | 239 G/A (codon 57)  A to C | Gly57Glu |  |  |

**Supplemental Table 2: Comparison of Baseline Characteristics between Cases and Controls**

| **Baseline characteristics** | **Cases [%] n=36** | **Controls [%], n=82** | **p-value** |
| --- | --- | --- | --- |
| Male | 16[44.4] | 41[50] | 0.578 |
| Religion  Hindu | 19[52.8] | 47[57.3] | 0.900 |
| Christian | 1[2.8] | 2[2.4] |  |
| Muslim | 16[44.4] | 33[40.2] |  |
| Low Birthweight | 1[2.8] | 14[17.1] | **0.036*** |
| WAZ [<-2SD]  At 6 months | 8[22.2] | 33[40.2] | 0.058 |
| At 12 months | 18[50] | 43[53.1] | 0.758 |
| At 24 months | 23[63.9] | 56[68.3] | 0.640 |
| HAZ [<-2SD]  At 6 months | 11[30.5] | 33[40.2] | 0.316 |
| At 12 months | 9[25] | 31[37.8] | 0.176 |
| At 24 months | 14[38.9] | 39[47.5] | 0.383 |
| Socioeconomic status**  Low | 21[58.3] | 49[59.7] | 0.274 |
| Middle | 15[41.7] | 28[34.2] |  |
| High | 5[6.1] | 5[6.1] |  |
| Number of diarrheal episodes [Median] | 6.5[5-9] | 2[1-3] | **0.0001** |

Fishers’ exact test; *Mann-Whitney U tests ** assessed using the Modified Kuppuswamy scale ^42^
